# Evidence for Transitional Stages in the Evolution of Euglenid Group II Introns and Twintrons in the *Monomorphina aenigmatica* Plastid Genome

**DOI:** 10.1371/journal.pone.0053433

**Published:** 2012-12-31

**Authors:** Jean-François Pombert, Erick R. James, Jan Janouškovec, Patrick J. Keeling

**Affiliations:** Department of Botany, University of British Columbia, Vancouver, British Columbia, Canada; University Of Montana - Missoula, United States of America

## Abstract

**Background:**

Photosynthetic euglenids acquired their plastid by secondary endosymbiosis of a prasinophyte-like green alga. But unlike its prasinophyte counterparts, the plastid genome of the euglenid *Euglena gracilis* is riddled with introns that interrupt almost every protein-encoding gene. The atypical group II introns and twintrons (introns-within-introns) found in the *E. gracilis* plastid have been hypothesized to have been acquired late in the evolution of euglenids, implying that massive numbers of introns may be lacking in other taxa. This late emergence was recently corroborated by the plastid genome sequences of the two basal euglenids, *Eutreptiella gymnastica* and *Eutreptia viridis*, which were found to contain fewer introns.

**Methodology/Principal Findings:**

To gain further insights into the proliferation of introns in euglenid plastids, we have characterized the complete plastid genome sequence of *Monomorphina aenigmatica*, a freshwater species occupying an intermediate phylogenetic position between early and late branching euglenids. The *M. aenigmatica* UTEX 1284 plastid genome (74,746 bp, 70.6% A+T, 87 genes) contains 53 intron insertion sites, of which 41 were found to be shared with other euglenids including 12 of the 15 twintron insertion sites reported in *E. gracilis*.

**Conclusions:**

The pattern of insertion sites suggests an ongoing but uneven process of intron gain in the lineage, with perhaps a minimum of two bursts of rapid intron proliferation. We also identified several sites that represent intermediates in the process of twintron evolution, where the external intron is in place, but not the internal one, offering a glimpse into how these convoluted molecular contraptions originate.

## Introduction

Euglenids are a morphologically diverse group of unicellular freshwater and marine eukaryotes displaying various feeding behaviours, including phagotrophy, osmotrophy, and phototrophy (reviewed in [1], [2]). Morphological and molecular phylogenetic inferences revealed that phototrophy emerged later in the evolution of this lineage ([2]–[4]) following the acquisition of a foreign plastid by secondary endosymbiosis from a green alga related to the free-living, deep-branching prasinophyte *Pyramimonas parkeae* [5]. The most well-known photosynthetic representative of euglenids is the freshwater flagellate *Euglena gracilis*, which has been used as a model organism for numerous studies in the past decades [6], [7].

One of the most remarkable features of the *E. gracilis* plastid genome (cpDNA) is the presence of over 150 introns (160 total) that interrupt almost all of its protein-coding genes (see [8] and GenBank X70810.2). Self-splicing catalytic ribozymes such as these are not uncommon in organelle genomes [9], [10], but the introns found in *E. gracilis* cpDNA are highly unusual. The majority of its introns are so-called group II introns, but they are often thoroughly reduced such that the normally conserved domains I to IV are absent or barely recognizable and, while the domain VI bulged adenosine required for the nucleophilic attack on the 5′ splice site of the intron has been retained, it is unclear whether these introns are still capable of self-splicing *in vivo*, or if they require the presence of an exogenous hydroxyl residue provided *in trans* [10], [11]. The *E. gracilis* plastid genome also contains several group III introns, a peculiar type of introns that are exclusive to the euglenid lineage. Group III introns are small U-rich highly degenerate group II introns from which they have retained only a modified 5′-NUNNG consensus and pseudo-domain VI in their 3′-end [12]. To complicate things further, the *E. gracilis* plastid genome also contains a number of twintrons (15 in total), which are introns inserted within other introns. These intricate nested introns must be removed sequentially to result in accurate splicing.

The unorthodox *E. gracilis* introns have been found in other *Euglena* species [13]–[15] including *Euglena* (formerly *Astasia*) *longa* [16], a close relative that has retained a plastid genome despite the loss of photosynthesis, but until recently it was not known if these peculiar genetic elements were representative of the euglenids as a whole. Early hypotheses postulated that these introns were acquired late in the evolution of photosynthetic euglenids [17] and the subsequent finding that the plastid genome of the prasinophyte *P. parkeae* did not contain any highly-reduced group II introns [5] gave further credence to the notion that these introns were gained after the secondary endosymbiotic plastid uptake. The recent characterisation of the complete plastid genome sequences from two early-diverging euglenids, *Eutreptiella gymnastica* [18] and *Eutreptia viridis* [19] revealed only 2 and 23 introns, respectively, also congruent with the intron-late hypothesis.

To better understand intron evolution in photosynthetic euglenids, we determined the complete plastid genome sequence of *Monomorphina aenigmatica* (strain UTEX 1284), a freshwater species that branches between *Euglena* and the earlier-diverging photosynthetic euglenids [20], [21]. The *M. aenigmatica* plastid genome (74,746 bp, 70.6% A+T, 87 genes) lends further support to the intron-late hypothesis, but also suggests that at least two distinct and independent intron acquisition bursts occurred in the euglenid plastid genomes. We also identify a number of insertion sites with an intermediate stage in the emergence of twintrons, showing how such molecular structures might arise.

## Results

**Figure 1:**
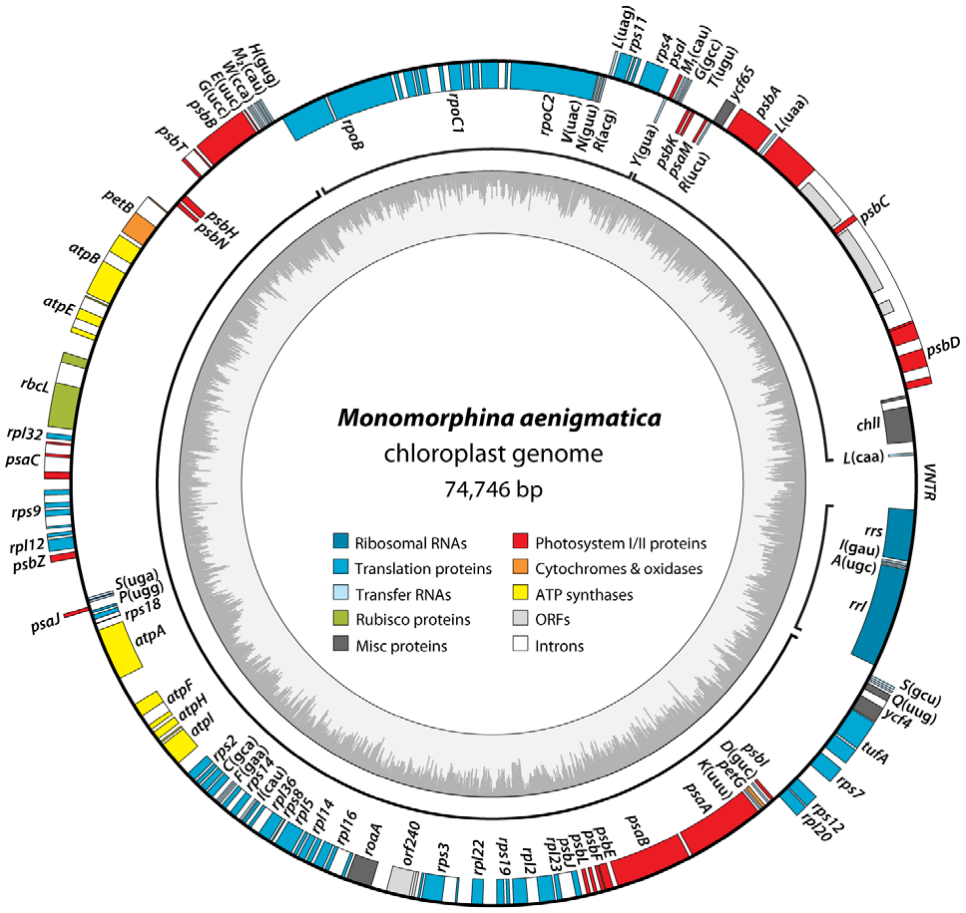
Gene map of the *Monomorphina aenigmatica* (UTEX 1284) plastid genome. Genes (filled boxes) located inside/outside the map are transcribed clockwise/counterclockwise. Introns are denoted by open boxes whereas intronic ORFs are illustrated as half-height boxes within the open boxes. tRNA genes are indicated by the one-letter amino acid code followed by the anticodon in parentheses. ORFs smaller than 150 amino acids are not shown. A+T/G+C content are shown in the inner circle in light and dark grey, respectively. Conserved gene clusters between *M. aenigmatica* and *E. gracilis* are denoted by brackets: *rpoB* to *Y*(gua), *M_1_*(cau) to *L*(caa), *rrs* to *rrl*, and *S*(gcu) to *H*(gug).

The chloroplast genome of *Monomorphina aenigmatica* strain UTEX 1284 maps as a circular molecule of 74,746 bp with an overall 70.6% A+T content (Figure 1, Table 1) and contains the usual complement of genes expected in photosynthetic algae. The *M. aenigmatica* cpDNA differs from its *E. gracilis* counterpart by only a few genes: it encodes the *psaI* gene missing from *E. gracilis*, but also lacks *psb30* (*ycf12*) and the gene coding for the small 5S rRNA, which are present in *E. gracilis*. The 5S rRNA-encoding gene is present in *Eta. viridis* but absent from *Etl. gymnastica* and, interestingly, from the prasinophytes *P. parkeae* and *Pycnococcus provasolii* [5].The highly divergent *rpoA* gene reported in some *Euglena* species [15] in-between *psbZ* and *trnS*(uga) does not appear to be present in *M. aenigmatica*. This location of the genome does not contain an ORF, and while the sequence shares barely-detectable similarity with the alpha subunit of bacterial DNA-directed RNA polymerases, it shares none with the reported euglenid RpoA sequences. The *M. aenigmatica* cpDNA contains only a single rRNA operon, which contrasts with the three copies found in tandem in the *E. gracilis* cpDNA. The 1∶1 relative coverage ratio between this operon and the other genomic regions in *M. aenigmatica* cpDNA assembly excludes the possibility that the single copy observed is the result of an assembly artefact that would have collapsed adjacent operons. Like its *E. gracilis* counterpart, however, the *M. aenigmatica* plastid genome features a number of tandem repeats restrained to a single locus, which are likely variable within the population. Interestingly, these variable numbers of tandem repeats (VNTR) are located in the same position, between *trnL*(caa) and *rrs*, as those reported in *E. gracilis*. The two genomes display a high level of synteny, with the 85 genes that they share distributed between four blocks of colinear genes.

**Table 1:** Main features of euglenid plastid DNAs and their closest known relative.

	*E. gracilis*	*E. longa*	*M. aenigmatica*	*Eta. viridis* f	*Etl. gymnastica*	*P. parkeae*
**A+T (%)**	73.9	77.6	70.6	71.4	65.7	65.3
**Size (bp)**						
Total	143,171	73,345	74,746	65,513	67,622	101,605
Genes^a^	62,776	49,860	45,568	44,061	50,573	80,191
Intergenic	24,712	11,357	13,048	9,288	10,176	18,657
Introns^b^	55,683	12,128	16,130	12,164	6,873	2,757
**Ratios (%)**						
Coding^a^	43.8	68.0	61.0	67.3	74.8	78.9
Intergenic	17.3	15.5	17.5	14.2	15.0	18.4
Intronic	38.9	16.5	21.6	18.6	10.2	2.7
**Genes** a,c						
Total	88^e^	57	87	84	86	110
Protein-coding	58^e^	27	58	56	58	81
tRNAs	27	27	27	25	26	27
rRNAs	3	3	2	3	2	2
**Intron insertion sites** d	139	60	53	23	7^h^	1
**Intron ORFs**	4	0	3	3	4	1
**rRNA operons**	3	3	1	≥2^g^	2	2

a Does not include intronic ORFs and pseudogenes.

b Includes intronic ORF.

c Duplicated genes were counted only once. Free-standing ORFs are not included.

d Twintrons (introns-within-introns) were counted as single insertion sites.

e Includes the *rpoA* gene reported in [15].

f The *orf103* annotated on the opposite strand of *rpl14*-*rpl16* in *Eta. viridis* [19] is spurious and was not taken into account.

g According to the read coverage described in Wiegert *et al*. [19].

h The *Etl. gymnastica* cpDNA features one intron in *atpA*, *rps2*, *rps18* and two introns in *psbC* that were not reported in Hrdá *et al*. [18] (see Data S1 for annotation).

Despite these similarities, the *M. aenigmatica* genome is roughly half the size of that of *E. gracilis* (Table 1). The two-fold difference in size between the two plastid genomes can be partly attributed to the three rRNA operons in *E. gracilis*, but is mostly due to a three-fold difference in the number of introns. Even the plastid genome of *E. longa* [16], a close non-photosynthetic relative of *E. gracilis*, encodes more introns than *M. aenigmatica*, despite its lower gene count due to the loss of photosynthesis. At the same time, however, the *M. aenigmatica* plastid contains more than twice the number of introns as *Eta. viridis* and over seven times the number as *Etl. gymnastica* [18], [19].

Of the 53 intron insertion sites we identified in *M. aenigmatica* cpDNA (Tables 1, 2, 3), 40 are shared with *E. gracilis* (Tables 2 and S1) [8]. The *M. aenigmatica* cpDNA also share a single cognate insertion site (*rpoC1*.Maen.6) with *E. longa* that is absent from *E. gracilis*, and which likely has been lost in the latter genome after the split with *E. longa*. Only one insertion site (*psbC*.Maen.1) was found to be shared between the four photosynthetic euglenids investigated to date (the *psbC* gene was lost in *E. longa*). The remaining 12 insertion sites in *M. aenigmatica* (Table 3) currently have no known homologs in sequenced euglenid or green algal plastid genomes, and broader homology searches based on the intron sequences did not identify potential homologs.

**Table 2:** Intron insertion sites in *M. aenigmatica* that are shared with other euglenid plastid DNAs.

Gene^a^	Shared insertion sitesa,b	Gene^a^	Shared insertion sitesa,b
***atpE***	**Maen.1, Egra.1**	*rpoC1*	Maen.8, Egra.9, Elon.9
*atpF*	Maen.1, Egra.2	***rpoC1***	**Maen.9, Egra.11, Elon.11**
*atpI*	Maen.1, Egra.6	*rps2*	Maen.1, Egra.1, Elon.1
*chlI*	Maen.1, Egra.1	*rps2*	Maen.2, Egra.2, Elon.2
***petB***	**Maen.1, Egra.1**	*rps2*	Maen.3, Egra.3, Elon.3
*psaC*	Maen.1, Egra.1	***rps3***	**Maen.1, Egra.1, Elon.1**
*psaC*	Maen.2, Egra.2	*rps3*	Maen.2, Egra.2, Elon.2
*psbB*	Maen.1, Egra.2	*rps8*	Maen.1, Egra.2, Elon.2
***psbC***	**Maen.1, Egra.2**	*rps8*	Maen.2, Egra.3, Elon.3
***psbC***	**Maen.2, Egra.4, Evir.1, Egym.1**	*rps9*	Maen.1, Egra.3
***psbK***	**Maen.1, Egra.2**	*rps9*	Maen.2, Egra.5, Elon.4
***psbT***	**Maen.1, Egra.1**	*rps9*	Maen.3, Egra.6, Elon.5
*rpl12*	Maen.1, Egra.1, Elon.1	*rps11*	Maen.1, Egra.1, Elon.1
*rpl14*	Maen.1, Egra.1, Elon.1	*rps11*	Maen.2, Egra.2, Elon.2
***rpl16***	**Maen.2, Egra.3, Elon.3**	*rps14*	Maen.1, Egra.1, Elon.1
***rpoC1***	**Maen.1, Egra.1, Elon.1**	*rps18*	Maen.1, Egra.1
*rpoC1*	Maen.2, Egra.2, Elon.2	***rps18***	**Maen.2, Egra.2, Egym.1** c
***rpoC1***	**Maen.3, Egra.3, Elon.3**	*rps19*	Maen.1, Egra.1, Elon.1
*rpoC1*	Maen.4, Egra.4, Elon.4	*tufA*	Maen.1, Egra.1, Elon.1
*rpoC1*	Maen.6, Elon.7	*ycf4*	Maen.1, Egra.1
*rpoC1*	Maen.7, Egra.8, Elon.8		

a Intron insertion sites reported to display twintrons in *Euglena gracilis* are highlighted in bold.

b Maen, *Monomorphina aenigmatica*; Egra, *Euglena gracilis*; Elon, *Euglena longa*; Evir, *Eutreptia viridis*; Egym, *Eutreptiella gymnastica*. The number corresponds to the insertion site on the gene (labelled from 5′ to 3′).

c The sequence of the *rps18*.Egym.1 intron is different from those in *M. aenigmatica* and *E. gracilis* and may be unrelated.

**Table 3:** Intron insertion sites unique to *M. aenigmatica*.

Unique insertion sites			
*atpB*.Maen.1	*psbD*.Maen.1	*rpl2*.Maen.1	*rpl23*.Maen.1
*atpE*.Maen.2	*psbD*.Maen.2	*rpl14*.Maen.2	*rpoB*.Maen.1^a^
*orf240*.Maen.1	*rbcL*.Maen.1	*rpl16*.Maen.1	*rpoC1*.Maen.5

a The *rpoB*.Maen.1 insertion site is located in the vicinity of *rpoB*.Evir.1, but it is unknown is these sites are related given the low conservation of the gene.

**Figure 2:**
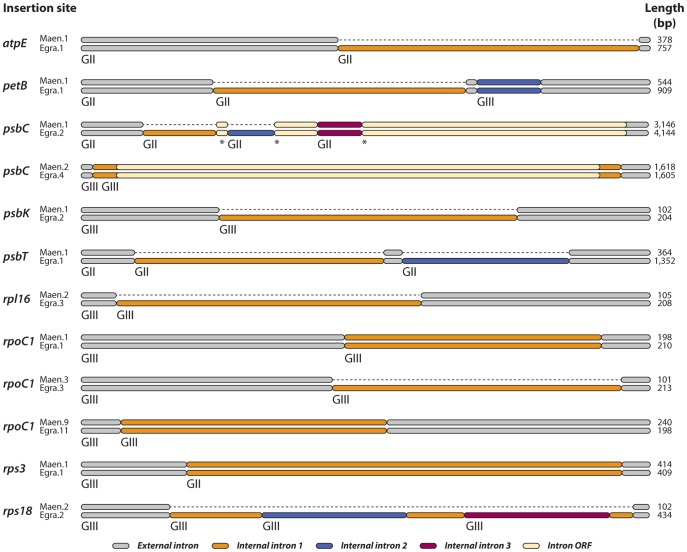
*Euglena gracilis* twintron insertion sites found in **M. aenigmatica**. Internal introns not found in *M. aenigmatica* are indicated by dashed lines. The total length of each insertion site is indicated on the right. Group II (GII) and group III (GIII) introns are indicated below the corresponding introns. Maen, *Monomorphina aenigmatica*; Egra, *Euglena gracilis*. The number after the period corresponds to the insertion site on the gene. In *psbC*.Egra.2, the fragmented ORF spliced together after excision of the internal introns is marked by asterisks.

Interestingly, 12 of the 15 sites reported to contain twintrons in *E. gracilis* also encode introns in *M. aenigmatica*. To determine whether twintrons were shared between *M. aenigmatica* and *E. gracilis*, we aligned the corresponding intron sequences at these positions (highlighted in Table 2) in two different ways. First, we aligned the corresponding *M. aenigmatica* sequence against all the twintrons from *E. gracilis*. Second, we aligned the *M. aenigmatica* sequence independently against each of the external and internal *E. gracilis* intron sequences. Furthermore, we also scanned the *M. aenigmatica* sequences for the 5′-NUNNG and abcdef(3-8)f′e′d′ A* c′b′a′ (4)-3′ consensuses described in [12]. The *M. aenigmatica* genome shares only 6 twintrons with *E. gracilis* (Figure 2). The *psbC*.Maen.2, *rpoC1*.Maen.2, *rpoC1*.Maen.9 and *rps3*.Maen.1 insertion sites display orthologs of the *E. gracilis* external and internal introns while, interestingly, *petB*.Maen.1 and *psbC*.Maen.1 contain the external and only one of the *E. gracilis* internal introns (the second and third twintron, respectively), probably reflecting intermediate stages of twintron evolution, although the possibility that the additional internal introns found in *E. gracilis* were lost in an ancestor of *M. aenigmatica* cannot be completely excluded. The six remaining insertion sites where *E. gracilis* encodes a twintron (*atpE*.Maen.1, *psbK*.Maen.1, *psbT*.Maen.1, *rpl16*.Maen.1, *rpoC1*.Maen.3, and *rps18*.Maen.2) were found to encode only a single intron in *M. aenigmatica*, and in all five cases the intron was orthologous to the *E. gracilis* external intron.

The *M. aenigmatica* chloroplast genome contains 3 putative reverse transcriptases/maturases. The first two are located within the intron insertion sites *psbC*.Maen.1 (*mat2*; *orf116* and *orf631*) and *psbC*.Maen.2 (*mat1*; *orf462*), whereas the third (*orf240*) is free-standing, potentially unique to *M. aenigmatica*, and highly degenerated (its putative inclusion within an unidentified intron, however, cannot be ruled out). The *mat1*/*mat2* intron-encoded maturases are not unique to *M. aenigmatica* and are also found in the other euglenid introns that are inserted at cognate sites (Table 2). In *E. gracilis*, the *mat2* segments corresponding to *M. aenigmatica*’s *orf116* and *orf631* form a single maturase after splicing of the internal introns [22], and we infer that this is also the case in *M. aenigmatica*. The other maturase, *mat1*, is highly conserved and found in many euglenids according to BLASTP searches.

## Discussion

**Figure 3:**
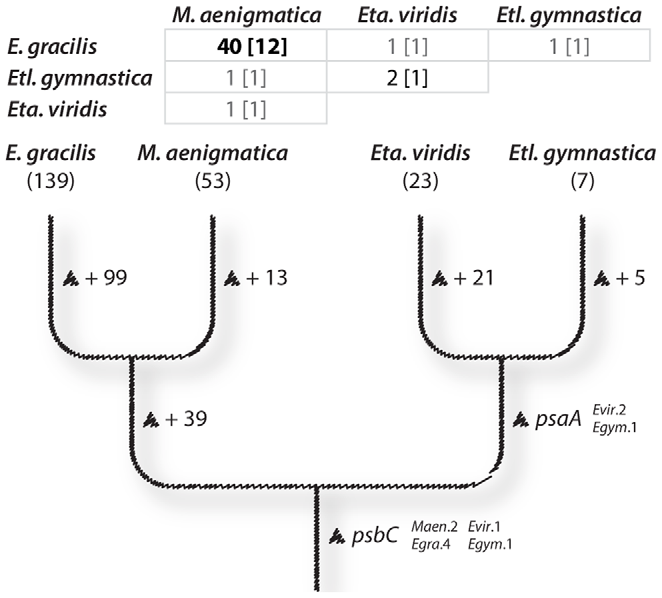
Phylogenetic mapping of intron insertion sites among euglenid chloroplast genomes. At the top is a matrix showing the number of insertion sites shared between pairwise taxa (shared twintron insertions sites are indicated between brackets). At the bottom is a cladogram showing the distribution of shared intron insertion sites across the euglenids. The total number of insertion sites for each species is indicated between parentheses below its name. Insertions across the phylogenetic tree are denoted by triangles. For this analysis, the ambiguous *rpoB* intron insertion sites were considered distinct. The no-longer-photosynthetic *E. longa* was not included in this figure due to its many gene losses. The phylogenetic relationships described here are schematized from Kim *et al*. [21].

All available evidence suggests that the introns found in euglenid cpDNAs were acquired after the secondary uptake of their plastid from a green alga. Neither their highly degenerate nor their canonical group II introns have been found in green algal plastid genomes to date. The few group II introns present in green algal plastids retain a canonical structure, and even the genome of their closest known free-living relative, *Pyraminomas parkeae*, features only a single intron that is unrelated to those found in euglenid cpDNAs. Furthermore, with 7 introns (and perhaps a few more left to identify), the genome of the early branching euglenid *Etl. gymnastica* [18] is relatively intron-poor, which corroborates the notion that their putative ancestor was also intron-poor. What is unclear however is how this remarkable expansion of intron numbers and alterations of their form took place in this lineage. Phylogenetic mapping of these insertion sites (Figure 3) suggests that the ancestral chloroplast genome already possessed the *psbC* intron found in all four photosynthetic euglenids, as proposed by Doetsch *et al.* [13], and that the other introns were mostly acquired later in euglenid evolution. Without additional density of sampling the possibility that the pattern of intron densities is a result of intron loss from an intron-rich common ancestor cannot be completely ruled out, but we find this explanation unlikely due to the low proportion of shared intron positions between distantly related taxa (Fig. 3). 75% of the *M. aenigmatica* insertion sites are shared with *E. gracilis* (and *E. longa*, when it still possesses the genes that harbour them), but 21 out of the 23 insertion sites found in *Eta. viridis* are currently unique to that species, and only one intron is common to all four species (Figure 3). Overall, the pattern suggests that intron acquisition was not a single massive wave, but partly due to a widespread ongoing gain of introns, and perhaps partly through at least two larger waves of acquisition that took place independently in different lineages (more sampling will be needed to test this possibility).

Twintrons are, by definition, introns-within-introns. This process implies an intermediate evolutionary stage in which the intron insertion site containing the twintron(s) featured only one intron, which eventually became the external intron in a twintron when a second intron was inserted within it. However, no such intermediate stage has ever been described, until now. *M. aenigmatica* contains twelve of the fifteen insertions sites known to encode twintrons in *E. gracilis*. In *M. aenigmatica*, half of these also encode twintrons whereas the other six sites contain only a single intron found to be orthologous to the *E. gracilis* external introns. These intermediate sites also give us better insights into the timeline of twintron arising in euglenid cpDNAs: we infer that the first twintron that appeared in euglenid plastid genomes is the one found in *psbC*, which is now found in *E. gracilis*, *M. aenigmatica*, *Etl. gymnastica*, *Eta. viridis* (Figure 3, Table 2) and other euglenids [13]. This putative ancestral twintron did not remain entirely static over time: the *Eta. viridis psbC* twintron is twice the size and contains two ORFs that appear to encode full-length maturases, suggesting that at least one other intron subsequently inserted itself within the *Eta. viridis* twintron. The other twintrons, absent from the *Eta. viridis* and *Etl. gymnastica* genomes, were likely gradually acquired in the branches leading to the ancestor of *Euglena* and *Monomorphina*, and the ancestor of *Euglena* itself. This process may still be ongoing, and it would be interesting to revisit *E. gracilis* strains whose generations have now been in culture for decades to see if we can detect significant changes.

Group II introns are mobile genetic elements known to interrupt target sequences, coding or non-coding. Introns that are inserted in intergenic regions are often transient owing to the relaxed selective pressure resulting from their not interrupting a coding sequence. In contrast, however, introns that are inserted within genes must be properly spliced for the gene to retain its function and any mutation affecting the splicing efficiency of the intron could have a deleterious effect. In twintrons these conditions are compounded, adding an extra layer of complexity to an already complex mechanism. It is therefore not surprising that twintrons are quite rare - other than euglenid plastids, a few isolated cases have been described (e.g. in a cryptomonad plastid [23], in the cyanobacterium *Thermosynechococcus elongatus* [24], in archaea [25], and in spliceosomal introns of *Drosophila* [26]). But the rarity of twintrons also raises the question, why does the *E. gracilis* plastid genome contain so many of them? The first explanation is perhaps a simple matter of density and mobility: in genomes with high intron mobility, more and more introns are expected to be found. This, in turn increases the likelihood of having an intron insert itself inside another intron. That being said, the *psbC* twintron cannot be explained in this way since it is present in all four euglenid genomes and likely arose before any intron proliferation. A second potential explanation is that group II intron mobility is often not random. Instead, they propagate at cognate and ectopic sites by processes known as ‘homing/retrohoming’ and ‘transposition/retrotransposition’, respectively. These processes are usually quite stringent, and while the endonucleases or reverse transcriptases involved allow for some polymorphism to occur, the target sites are usually highly conserved. If the target site is contained within a duplicated or repeated sequence however, then a given intron can, in theory, quickly move to occupy most of these loci. It is also possible that the transposition/retrotransposition processes in euglenid genomes are somehow relaxed, thus helping with the intron invasion. A third potential explanation is that in the euglenid plastid genetic system the selection for accurate splicing has been relaxed for some reason, allowing the proliferation of what might otherwise be slightly deleterious introns. It would be interesting to investigate whether the transcription is generally messy in these plastid genomes, and if the mRNAs for intron-interrupted genes form a population of viable and defective molecules. Since previous analyses found a number of splicing variants in *E. gracilis* [27], [28], this certainly appears plausible.

### Conclusions

Almost 20 years have passed since the sequencing of the first euglenid plastid genome revealed its unusual introns, but our density of sampling is only now starting to approach the level where we can see patterns that allow us to reconstruct the events that led to their remarkable characteristics. We can now start to see how the density of introns has grown in a lineage specific manner, and how the stepwise assembly of twintrons proceeded through the characterization of genomes that retain intermediate stages of these processes. Plastid genomes from several euglenid families are still unrepresented, however, so hopefully in the near future even more density of sampling across the tree of euglenids, but also from groups of closely related strains will become available. Both approaches potentially hold a wealth of information about the euglenid introns and additional clues into the evolution of these unorthodox genetic elements.

## Methods

### Strain, Culture and Nucleic Acids Extraction

*Monomorphina aenigmatica* strain UTEX 1284 was obtained from the University of Texas culture collection under the name *Phacus megalopsis*. Previous studies [29] show that this specific culture is not *P. megalopsis* but rather *M. aenigmatica*, and the 18S sequence we obtained from the illumina sequencing described below confirms that this is a *Monomorphina* species. The *M. aenigmatica* UTEX 1284 starter culture was grown in Bold 3N medium at 20°C with 12∶12 hours light:dark cycles for two months. Cells were pelleted at maximum speed in an Eppendorf 5810 R centrifuge and the supernatant medium discarded. The pelleted cells were ground with a mortar and pestle in the presence of liquid nitrogen, and total DNA extracted from the resulting powder with the MasterPure DNA extraction kit (Epicentre) using the protocol provided by the manufacturer. Polysaccharides were removed from the extracted DNA as follows. Total DNA (50 µl) was incubated in the presence of 500 µl CTAB buffer (2% CTAB, 100 mM Tris-Hcl, 20 mM EDTA, 1.4 M NaCl, 0.2% β-mercaptoethanol, 0.1 mg/ml proteinase K) at 60°C, cleaned with 500 µl of chloroform, precipitated with 500 µl of isopropanol, washed with 500 µl of EtOH 80% (vol/vol), then resuspended in water. Total RNA was extracted from liquid NO_2_-ground and pelleted cells with Trizol (Invitrogen) using the protocol provided by the manufacturer.

### DNA Sequencing and Assembly

The *M. aenigmatica* illumina TruSeq library (100 bp paired-ends, 220 bp insert size) was prepared from total DNA and sequenced on one lane of illumina HiSeq by the Beaty Biodiversity Research Centre sequencing facilities (Vancouver, Canada). Adapters and indexes were removed from the resulting reads and the reads filtered using the CASAVA 1.8.2 pipeline (illumina) under the default parameters. The first 320,000,000 reads (16,000,000 pairs) of the filtered reads were assembled in parallel with Ray version 2.0.0 rc4 [30] and a k-mer value of 21, using 256 processing cores (32 Intel Nehalem CPUs @ 2.8 Ghz) distributed between 16 processing nodes on the Colosse (CLUMEQ, ComputeCanada) with a maximum RAM allowance of 384 Gb. The 1,849,947 contigs thus generated (546,420,088 bp total) were filtered by size and those equal to or larger than 500 bp (130,292 contigs, 241,607,848 bp total) were used for downstream analyses. Contigs from the *M. aenigmatica* plastid genome were identified as follows. The 500+ bp-filtered contigs were converted to a BLAST [31] database using makeblastdb from the NCBI BLAST package version 2.2.26. Then, the protein set of *Euglena gracilis* [EMBL:X70810] and *Pyramimonas parkeae* [GenBank:NC_012099] were both queried independently against the *M. aenigmatica* database with TBLASTN from the BLAST package using variable cut-offs to detect potential chloroplast contigs. The potential contigs of interest were pulled out from the multifasta file with the faOneRecord/faSomeRecords command line utilities from the University of California, Santa Cruz (UCSC), and searched reciprocally against the entire NCBI non-redundant database to ascertain their origin.

The *M. aenigmatica* chloroplast contigs were further assembled and polished with CONSED 23 [32]. To do so, subsets of the illumina reads were iteratively mapped back on the contigs with the addSolexaReads.pl script from the CONSED package, modified to increase the mapping stringency (i.e. -minmatch 50, -minscore 50, -penalty −9). Contigs were extended according to the paired-ends information, linked, and verified by mapping back the reads on the resulting assemblies. The overall coverage across the genome was then assessed to detect the presence of assembly artefacts potentially caused by repeated/duplicated regions differing from a 1∶1 coverage ratio. To do so, reads were mapped on the final assembly (780 X sequencing depth) with Bowtie 0.12.8 [33], and the assembly visually inspected with Tablet 1.12 [34].

### Genome Annotation

Genes coding for tRNAs were identified with tRNAscan-SE 1.21 [35] while rRNA-encoding genes were identified by BLAST homology searches [31] using orthologs from the *E. gracilis* genome as input queries. Protein-coding genes and open reading frames were annotated with Artemis 14.0 using the built-in tools. Then, each predicted *M. aenigmatica* protein-coding gene sequence was aligned at the amino acid level against orthologs from euglenids and green algae using the L-INS-i algorithm from MAFFT 6.925 [36] to detect missing exons and/or erroneous exon-intron boundaries. Genes potentially absent from the *M. aenigmatica* plastid genome were searched for specifically by BLAST homology searches using the closest known orthologs. The predicted exon-intron junctions were annotated to preserve homology between orthologs whenever possible. The gene map shown in Figure 1 was generated with OGDraw 1.2 [37] and refined manually. The *M. aenigmatica* UTEX 1284 plastid genome annotation has been deposited in GenBank [GenBank:JX457480].

### RT-PCRs

Because the *rpoC1* gene is not well conserved, we performed RT-PCR experiments using primers specific to *M. aenigmatica* to ascertain a number of intron-exon boundaries. The RT-PCRs were performed on total RNA with the Superscript III One-Step RT-PCR kit (Invitrogen) and the resulting products cloned with the StrataClone PCR cloning kit (Stratagene), both using the protocols provided by the manufacturers. The clones grown on LB-ampicillin (50 µg/ml) plates with X-galactose (20 mg/ml) at 37°C overnight were PCR-screened for inserts using the T3/T7 primers flanking the insertion site and the EconoTaq PLUS GREEN (Lucigen) and plated again on LB+ampicillin (37°C/8 hours). Selected clones were cultured overnight at 37°C in 2 ml of liquid LB medium+ampicillin (50 µg/ml) and the plasmids purified with the FastPlasmid Mini kit (5 Prime) using the default protocol. The products were then Sanger-sequenced using the T3/T7 primers flanking the insertion site. Note that we could not produce a reliable RT-PCR covering the insertion site located at position 4 in *M. aenigmatica*’s rpoC1, and believe that a small unannotated exon might be present within it.

### Intron Analysis

Cognate intron insertion sites between euglenid genomes (*M. aenigmatica* [GenBank:JX457480], *E. gracilis* [EMBL:X70810], *E. longa* [EMBL: AJ294725], *Eta. viridis* [GenBank:JN643723], *Etl. gymnastica* [EMBL:HE605038]) were determined as follows. Orthologous protein sequences were aligned with their green algal counterparts using the L-INS-i algorithm from MAFFT and the insertion sites mapped manually on the corresponding sequences. Insertions located within the same amino acid but at different sites within the corresponding codon were considered distinct. We also corrected a number of annotations from the *Eta. viridis* and *Etl. gymnastica* accessions numbers (Data S1) for which exon-intron boundaries were erroneous. For *Etl. gymnastica*, we further annotated five additional introns that were not in the original accession number (Data S1). Introns inserted at cognate sites were aligned with MAFFT-L-INS-i and the presence of shared twintrons detected by 1) aligning the full span of the twintrons from *E. gracilis* against the target sequence and 2) aligning each of the external and internal *E. gracilis* intron sequences against the target sequence. For *M. aenigmatica* sequences, we also scanned for the 5′-NUNNG and abcdef(3–8)f′e′d′ A* c′b′a′ (4)-3′ consensuses described in [12] whenever a twintron was suspected, to insure that the increased size was not due to an intron that was simply longer than usual.

## Supporting Information

Table S1 **Intron insertion sites in the photosynthetic plastid genomes of the euglenids** ***E. gracilis*** **,** ***M. aenigmatica*** **,** ***Eta. viridis*** **and** ***Etl. gymnastica*** **.** The twintron insertion sites in *E. gracilis* are highlighted in blue. Insertions sites in *Eta. viridis* and *Etl. gymnastica* are predictions only and therefore subject to change. The *psbD*.Egra.10 and *psbC*.Egra.1 insertion sites are the same (they are located in the overlap between the two genes). The *rpoC1*.Maen.4 insertion could not be verified by RT-PCR and may contain a short additional exon.
(XLSX)

Data S1 **Euglenid plastid genomes EMBL annotations files.** The *Eta. viridis* and *Etl. gymnastica* original annotations were modified to include additional introns and/or revised insertions sites. The modifications are also indicated by miscellaneous features annotation tags in the revised files for quick localization with Artemis.
(ZIP)

## References

[1] Leander BS, Esson HJ, Breglia SA (2007) Macroevolution of complex cytoskeletal systems in euglenids. BioEssays 29: 987–1000. 17876783 10.1002/bies.20645

[2] Leander BS (2004) Did trypanosomatid parasites have photosynthetic ancestors? Trends in Microbiology 12: 251–258. 15165602 10.1016/j.tim.2004.04.001

[3] Leander BS, Triemer RE, Farmer MA (2001) Character evolution in heterotrophic euglenids. European Journal of Protistology 37: 337–356.

[4] Breglia SA, Slamovits CH, Leander BS (2007) Phylogeny of phagotrophic euglenids (Euglenozoa) as inferred from *hsp90* gene sequences. Journal of Eukaryotic Microbiology 54: 86–92. 17300525 10.1111/j.1550-7408.2006.00233.x

[5] Turmel M, Gagnon M-C, O’Kelly CJ, Otis C, Lemieux C (2009) The chloroplast genomes of the green algae *Pyramimonas*, *Monomastix*, and *Pycnococcus* shed new light on the evolutionary history of prasinophytes and the origin of the secondary chloroplasts of euglenids. Molecular Biology and Evolution 26: 631–648. 19074760 10.1093/molbev/msn285

[6] Hedges SB (2002) The origin and evolution of model organisms. Nature Reviews Genetics 3: 838–849. 10.1038/nrg929 12415314

[7] Krajčovič J, Ebringer L, Schwartzbach SD (2002) Reversion of endosymbiosis? The case of bleaching in *Euglena*. In: Seckbach J, editor. Symbiosis: Mechanisms and Model Systems. Dordrecht: Kluwer Academic Publishers. 185–206.

[8] Hallick RB, Hong L, Drager RG, Favreau MR, Monfort A, et al (1993) Complete sequence of *Eugena gracilis* chloroplast DNA. Nucleic Acids Research 21: 3537–3544. 8346031 10.1093/nar/21.15.3537 PMC331456

[9] Cech TR (1990) Self-splicing of group I introns. Annual Review of Biochemistry 59: 543–568. 10.1146/annurev.bi.59.070190.002551 2197983

[10] Lambowitz AM, Zimmerly S (2011) Group II introns: mobile ribozymes that invade DNA. Cold Spring Harbor Perspectives in Biology 3: a003616. 20463000 10.1101/cshperspect.a003616 PMC3140690

[11] Lambowitz AM, Zimmerly S (2004) Mobile group II introns. Annual Review of Genetics 38: 1–35. 10.1146/annurev.genet.38.072902.091600 15568970

[12] Copertino DW, Hallick RB (1993) Group II and group III introns of twintrons: potential relationships with nuclear pre-mRNA introns. Trends in Biochemical Sciences 18: 467–471. 8108859 10.1016/0968-0004(93)90008-b

[13] Doetsch NA, Thompson MD, Hallick RB (1998) A maturase-encoding group III twintron is conserved in deeply rooted euglenoid species: are group III introns the chicken or the egg? Molecular Biology and Evolution 15: 76–86. 9491607 10.1093/oxfordjournals.molbev.a025850

[14] Doetsch NA, Thompson MD, Favreau MR, Hallick RB (2001) Comparison of *psbK* operon organization and group III intron content in chloroplast genomes of 12 Euglenoid species. Molecular and General Genetics 264: 682–690. 11212923 10.1007/s004380000355

[15] Sheveleva EV, Giordani NV, Hallick RB (2002) Identification and comparative analysis of the chloroplast alpha-subunit gene of DNA-dependent RNA polymerase from seven *Euglena* species. Nucleic Acids Research 30: 1247–1254. 11861918 10.1093/nar/30.5.1247 PMC101230

[16] Gockel G, Hachtel W (2000) Complete gene map of the plastid genome of the nonphotosynthetic euglenoid flagellate *Astasia longa* . Protist 151: 347–351. 11212895 10.1078/S1434-4610(04)70033-4

[17] Thompson MD, Copertino DW, Thompson E, Favreau MR, Hallick RB (1995) Evidence for the late origin of introns in chloroplast genes from an evolutionary analysis of the genus *Euglena* . Nucleic Acids Research 23: 4745–4752. 8532514 10.1093/nar/23.23.4745 PMC307460

[18] Hrdá Š, Fousek J, Szabová J, Hampl V, Vlček Č (2012) The plastid genome of *Eutreptiella* provides a window into the process of secondary endosymbiosis of plastid in euglenids. PloS ONE 7: e33746. 22448269 10.1371/journal.pone.0033746 PMC3308993

[19] Wiegert KE, Bennett MS, Triemer RE (2012) Evolution of the chloroplast genome in photosynthetic euglenoids: A comparison of *Eutreptia viridis* and *Euglena gracilis* (Euglenophyta). Protist 163: 832–843. 22364772 10.1016/j.protis.2012.01.002

[20] Linton EW, Karnkowska-Ishikawa A, Kim JI, Shin W, Bennett MS, et al (2010) Reconstructing euglenoid evolutionary relationships using three genes: nuclear SSU and LSU, and chloroplast SSU rDNA sequences and the description of *Euglenaria* gen. nov. (Euglenophyta). Protist 161: 603–619. 20434949 10.1016/j.protis.2010.02.002

[21] Kim JI, Shin W, Triemer RE (2010) Multigene analyses of photosynthetic euglenoids and new family, *Phacaceae* (Euglenales). Journal of Phycology 46: 1278–1287.

[22] Zhang L, Jenkins KP, Stutz E, Hallick RB (1995) The *Euglena gracilis* intron-encoded *mat2* locus is interrupted by three additional group II introns. RNA 1: 1079–1088. 8595563 PMC1369334

[23] Maier UG, Rensing SA, Igloi GL, Maerz M (1995) Twintrons are not unique to the *Euglena* chloroplast genome: structure and evolution of a plastome *cpn60* gene from a cryptomonad. Molecular & General Genetics 246: 128–131. 7823908 10.1007/BF00290141

[24] Mohr G, Ghanem E, Lambowitz AM (2010) Mechanisms used for genomic proliferation by thermophilic group II introns. PLoS Biology 8: e1000391. 20543989 10.1371/journal.pbio.1000391 PMC2882425

[25] Dai L, Zimmerly S (2003) ORF-less and reverse-transcriptase-encoding group II introns in archaebacteria, with a pattern of homing into related group II intron ORFs. RNA 9: 14–19. 12554871 10.1261/rna.2126203 PMC1370365

[26] Scamborova P, Wong A, Steitz JA (2004) An intronic enhancer regulates splicing of the twintron of *Drosophila melanogaster prospero* pre-mRNA by two different spliceosomes. Molecular and Cellular Biology 24: 1855–1869. 14966268 10.1128/MCB.24.5.1855-1869.2004 PMC350559

[27] Drager RG, Hallick RB (1993) A complex twintron is excised as four individual introns. Nucleic Acids Research 21: 2389–2394. 7685079 10.1093/nar/21.10.2389 PMC309537

[28] Jenkins KP, Hong L, Hallick RB (1995) Alternative splicing of the *Euglena gracilis* chloroplast *roaA* transcript. RNA 1: 624–633. 7489521 PMC1369306

[29] Nudelman MA, Leonardi PI, Conforti V, Farmer MA, Triemer RE (2006) Fine structure and taxonomy of *Monomorphina aenigmatica* comb. nov. (Euglenophyta). Journal of Phycology 42: 194–202. 27040898 10.1111/j.1529-8817.2006.00170.x

[30] Boisvert S, Laviolette F, Corbeil J (2010) Ray: Simultaneous assembly of reads from a mix of high-throughput sequencing technologies. J Comput Biol 17: 1519–1533. 20958248 10.1089/cmb.2009.0238 PMC3119603

[31] Altschul SF, Gish W, Miller W, Myers EW, Lipman DJ (1990) Basic Local Alignment Search Tool. J Mol Biol 215: 403–410. 2231712 10.1016/S0022-2836(05)80360-2

[32] Gordon D, Abajian C, Green P (1998) Consed: a graphical tool for sequence finishing. Genome Res 8: 195–202. 9521923 10.1101/gr.8.3.195

[33] Langmead B, Trapnell C, Pop M, Salzberg SL (2009) Ultrafast and memory-efficient alignment of short DNA sequences to the human genome. Genome Biol 10: R25. 19261174 10.1186/gb-2009-10-3-r25 PMC2690996

[34] Milne I, Bayer M, Cardle L, Shaw P, Stephen G, et al (2010) Tablet - next generation sequence assembly visualization. Bioinformatics 26: 401–402. 19965881 10.1093/bioinformatics/btp666 PMC2815658

[35] Lowe TM, Eddy SR (1997) tRNAscan-SE: a program for improved detection of transfer RNA genes in genomic sequence. Nucleic Acids Res 25: 955–964. 9023104 10.1093/nar/25.5.955 PMC146525

[36] Katoh K, Toh H (2010) Parallelization of the MAFFT multiple sequence alignment program. Bioinformatics 26: 1899–1900. 20427515 10.1093/bioinformatics/btq224 PMC2905546

[37] Lohse M, Drechsel O, Bock R (2007) OrganellarGenomeDRAW (OGDRAW): a tool for the easy generation of high-quality custom graphical maps of plastid and mitochondrial genomes. Current Genetics 52: 267–274. 17957369 10.1007/s00294-007-0161-y

